# Supplementary material for: Digital economy, innovation factor allocation and industrial structure transformation—A case study of the Yangtze River Delta city cluster in China
Source: PLoS One. 2024 Apr 10;19(4):e0300788. doi: 10.1371/journal.pone.0300788 (PMC11006168; doi:10.1371/journal.pone.0300788)
Supplement: Attachment — Submitted filename: Response to Reviewers.docx [file pone.0300788.s002.docx]

**Response to reviewers**

Dear reviewers:

We feel great thanks for your professional review work on our article. As you are concerned, there are several problems that need to be addressed. According to your nice suggestions, we have made extensive corrections to our previous draft, the detailed corrections are listed below.

To facilitate this discussion, we first retype your comments in italic font and then present our responses to the comments.

**Reviewer #1:**

**Comment1:** *Abstract is a little longer, please make it shorter and show more about the important findings to readers.*

**Response1：**Dear reviewers, thank you very much for your careful review and valuable suggestions. First of all, we have improved the abstract section according to your suggestions, i.e. streamlined the language of the research conclusion that ' digital economy has significantly promoted industrial structure transformation '. Combined with Opinion 7, the content of panel effect is deleted, and the mediating model is selected for discussion to make the paper much clearer to discuss one topic, thank you again for your valuable comments.

**Comment2:** *In the first paragraph of Introduction, the author discussed a lot of service. It is very strange for us as a reader why you discuss service here. I can understand you when I read the measurement of industrial structure using service. My suggestion is to write more about the important of the role of digital economy in industrial structure upgrade. it will be better not to mention some new terms (or some second-level constructs) not relevant to the title.*

**Response2:** Dear reviewers, thank you very much for your careful review and valuable suggestions. We have revised the content of the introduction according to your suggestions, focusing on the background of industrial structure transformation, and the role of digital economy in industrial structure transformation. The specific concepts and different dimensions of industrial structure transformation are introduced in section 2, and thank you again for your valuable comments.

**Comment3:** *I suggest you not use Political person or political conference to prove the importance. it is better to use data, evidence, or by citing some importance references to discuss the importance. in the paper, you discuss Xi jinping, 20th party congress. All these citations are not academic style.*

**Response3:** Thank you again for your valuable suggestions for the paper. We have shifted the way to express the importance, and added relevant content to the paper, adding a discussion about the status of the Yangtze River Delta urban agglomeration in the international as well as Chinese domestic context. We also express the population, gross product and land area of the Yangtze River Delta city cluster.

**Comment4:** *Introduction part is too long. I suggest you reduce it into 1.5-2 pades. Please mention research gaps in literature review and give your contributions.*

**Response4:** Dear reviewers, thank you very much for your careful review and valuable suggestions. Taking account to your comments and those of another reviewer, in order to make the structure of the paper clearer, we have deleted the content related to service and compressed content to make the structure of the paper smoother. The research gaps and contributions are also determined accordingly in the later.

**Comment5:** *The Plos one is a well-established international journal. I find author cite many references writing in Chinese. Please cut them or replace them with some relevant and good journal papers.By the way, the format should fit to Plos one style or international style like APA, Chicago and so on. the [J] style is Chinese standard, not an international one. I hope you can understand me and I did not spell them out here. Further, I find most authors of your references are Chinese writers. Is any non-Chinese background scholars write this kind of papers? Please do the systematic review first.*

**Response5:** Thank you again for your valuable suggestions for the paper. We have cut the references writing in Chinese and added relevant good journal papers and content to the paper, adding a discussion of relevant research conducted in other countries on the digital economy and industrial structure transformation. We have changed the reference format to APA format.

**Comment6:** *The English should be substantially improved by a native speaker service or by some professionals. the following is some examples,in abstract, Digital economy empowering industrial structure transformation is crucial in achieving regional high-quality development. This sentence can be changed into, The attainment of regional high-quality development necessitates the critical role of the digital economy in facilitating the transformation of industrial structures. or The restructuring of the industrial structure, enabled by the digital economy, is essential to attaining high-quality growth in the area.*

**Response6:** Thank you for your valuable and thoughtful comments. We have carefully checked and improved the English writing in the revised manuscript. Please see if the revised version met the English presentation standard.

**Comment7:** *the authors use mediating model and also use threshold effect model. I suggest you use one of them to make the paper much clearer to discuss one topic and also make the paper shorter and readable. No one can talk all the story in one paper. There are ten tables in the text, so many.*

**Response7:** Thank you again for your valuable suggestions for the paper. Based on your suggestions and after consideration, we decided to delete the analysis of the threshold effect section, focus on the mediating effect, and add content description in the empirical and conclusion sections to make the article look clearer.

**Comment8:** *Section 6 is not good. I can translate your text into 'our research method (sample) has problem; we can also add spillover effect but we refuse to do so.' All these discussions are not good to accept this paper. I suggest you think about new directions based on this paper, and combine this section within conclusion part Section 5. do not write a new part.*

**Response8:** Dear reviewers, thank you very much for your careful review and valuable suggestions. Considering your suggestions, we put the research limitations and prospects in the Section 5, and elaborate from the perspective of Index construction and measurement, thank you again for your valuable comments

**Reviewer #2**

**Comment1:** *The introduction of research background and significance is not clear.*

**Response1:** Thanks for your great suggestion on improving the accessibility of our manuscript. We reinterpreted the research background and significance, mainly increasing the role of digital economy in the transformation of industrial structure, and using data, evidence to discuss the importance.

**Comment2:** *The author should summarize the existing research gaps and highlight the* *innovation of this paper after completing the literature review.*

**Response2:** Thanks for your valuable comments on the manuscript. First, we summarize the research gap from three aspects: research perspective, index measurement and research area. Second, the innovation of this paper is put forward in correspondence with the research gaps, we further elaborate on the differences and novelty of the results of the study through the Yangtze River Delta city cluster from previous studies in the context of possible marginal contributions.

**Comment3:** *The author needs to introduce the reasons for the research method and combine it with the innovation of the research in this paper.*

**Response3:** Dear reviewers, thank you very much for your careful review and valuable suggestions. First, it is the innovation of this paper to measure the transformation of industrial structure from four dimensions. Therefore, the connotation of industrial structure transformation is explained at the beginning of theoretical analysis, which is also the basis of index measurement. We supplement the reasons for the selection of methods and reflect them in research innovation. Second, the entropy method calculates the core explanatory variable digital economy, so we have added the introduction of this method in the index interpretation section.

**Comment4:** *This article has obtained some interesting findings through the models, but these findings need to be further verified from theory or actual conditions. Also, further highlight the contribution of this article.*

**Response4:** Thanks for your valuable comments on the manuscript. After getting some findings of the empirical part, we add the theoretical explanation of the conclusion and the comparison with the previous scholars ' research results in the part of benchmark regression and intermediary effect test, we also complement the discussion section and highlight the contributions of this article.

**Comment5:** *The discussion should focus on describing the differences between the article study and other scholars' studies, thus highlighting the relevance and academic value of the article, the following literature should be helpful for your research：(1) Reduction pathways identification of Agricultural Water Pollution in Hubei Province, China. (2) A differential game of water pollution management in the trans-jurisdictional river basin.*

**Response5:** Dear reviewers, thank you very much for your careful review and valuable suggestions. It is really true as reviewer suggested that the discussion should focus on describing the differences between article research and other scholars, so we refer to the literature you have given, and modify the literature review and results discussion section in combination with their contents and methods.

We would like to take this opportunity to thank you for all your time involved and this great opportunity for us to improve the manuscript. We hope you will find this revised version satisfactory.

Yours sincerely,

The Authors
